# The “Utility” of Highly Toxic Marine-Sourced Compounds

**DOI:** 10.3390/md17060324

**Published:** 2019-05-31

**Authors:** David J. Newman

**Affiliations:** Newman Consulting LLC, Wayne, PA 19087, USA; djnewman664@verizon.net; Tel.: +1-610-971-9784

**Keywords:** antibody-drug-conjugates, marine-sourced, toxic-compounds, cancer, clinical trials

## Abstract

Currently a few compounds isolated from marine sources have become drugs, mainly directed towards cancer and pain. Compounds from marine sources have exquisite potencies against eukaryotic cells, as they act as protective agents against attack by predators in the marine environment. Their toxicities act as a “double-edged sword” as they are often too toxic for direct use in humans and thus have to be chemically modified. By linking suitably modified compounds to monoclonal antibodies directed against specific epitopes in mammalian cancer cells, they can be delivered to a specific cell type in humans. This review updates and extends an article published in early 2017, demonstrating how by careful chemical modifications, highly toxic compounds, frequently peptidic in nature, can be utilized as antitumor drug candidates. The antibody-drug- conjugates (ADCs) discussed are those that are currently in clinical trials listed in the NIH Clinical Trials Registry as, “currently active, recruiting or in some cases, recently completed”. There are also some ADCs discussed that are at the advanced preclinical stage, that in some cases, are repurposing current drug entities, and the review finishes with a short discussion of the aplyronines as potential candidate warheads as a result of scalable synthetic processes.

## 1. Introduction

Currently the following small molecule marine-sourced/derived compounds are in clinical use in diseases related to cancer, in a variety of countries, including the US and Australia, and the EU: Ecteinascidin 743 (Yondelis^®^) approved in the EU in 2007; Eribulin (Halaven^®^) approved in the US in 2010 and Aplidine (Plitidepsin^®^) approved in Australia in 2018, with the first “direct from the sea” agent, ziconotide (Prialt^®^) approved in the US for intractable pain in 2004.

However, readers might ask about brentuximab vedotin (Adcetris^®^) which was approved by the US FDA in 2011 as a treatment for lymphoma. This compound was deliberately not placed in the paragraph above, as it is entirely different “pharmacologically” to those compounds. It is composed of a highly toxic “warhead” derived from dolastatin 10 attached to a specific targeting moiety, a monoclonal antibody (mAb) directed to a particular epitope on a cancer cell. Full details are presented below in the relevant section.

This review is an updated version of one published in *Marine Drugs* in 2017 [1] and though there obviously will be duplication due to the time that clinical trials take, particularly in cancer, this review still covers the warheads used and some of their history, as the majority have come from the pioneering work of the Pettit group on the dolastatins and their chemical descendants. It covers antibody drug conjugates (ADCs) that have been approved, are in the post-New Drug Application (NDA) process, are in clinical trials, or are in advanced preclinical stages of evaluation as of the middle of April 2019. Rather than give simple lists of trade names and “warheads”, they are subdivided by clinical stage and further subdivided by “warhead”. In each case, the total number(s) of trials will not be given, which is different from the 2017 review, and the current review concentrates on those that are currently underway at the recruiting or active stages, or in a few cases due to timing, the trials may have stopped recruiting or be at the stage of “completed” and awaiting publication and/or further trials at the same or different level. The clinical data were abstracted from the NIH clinical trials database which is a freely available asset, though in one case, data were abstracted from a report of trials in Japan (obtained from a search using Google).

This review cannot be a “complete” account, as frequently reports are made in the literature using only a code name for an ADC and without any information as to the “warhead”. An example is the ADC known as PF-06688992, which has as its warhead, “PF-06464368” whose identity is not available in the open literature. Thus, this review is as complete as it can be from using publicly available resources, but it does not contain every marine-derived warhead currently under trial.

There is also a discussion of a class of marine-sourced compounds, the aplyronines, that are extremely toxic agents with potential as warheads, but that are not as yet known to be attached to a suitable mAb. It will be seen that every ADC that is mentioned will have “cancer” as the potential target, due predominately to the source of funding for the marine-derived agents being the National Cancer Institute in the US. In other known examples, there are potential antibiotic agents delivered by the same process, but their source is not the marine environment, as yet!

## 2. The Evolution of Modified Dolastatins as ADC Warheads

**Figure 1:**
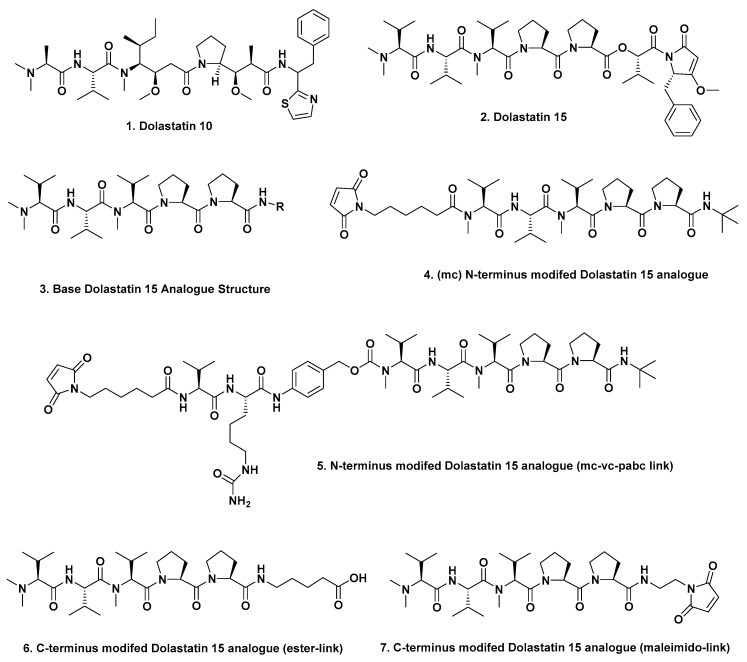
Structures **1** to **7**.

**Figure 2:**
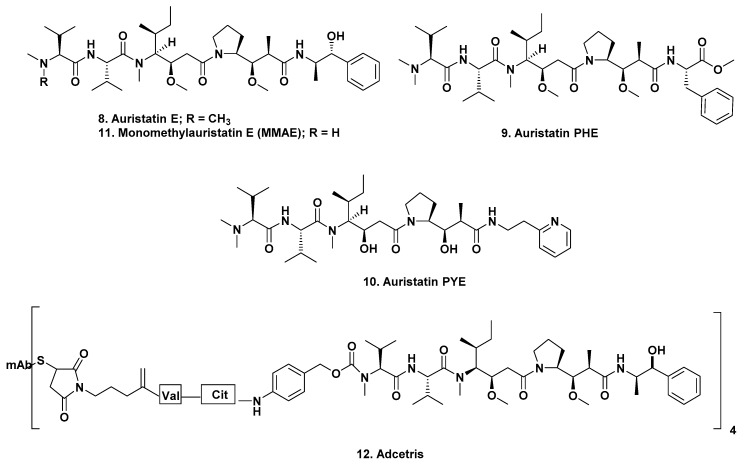
Structures **8** to **12**.

**Figure 3:**
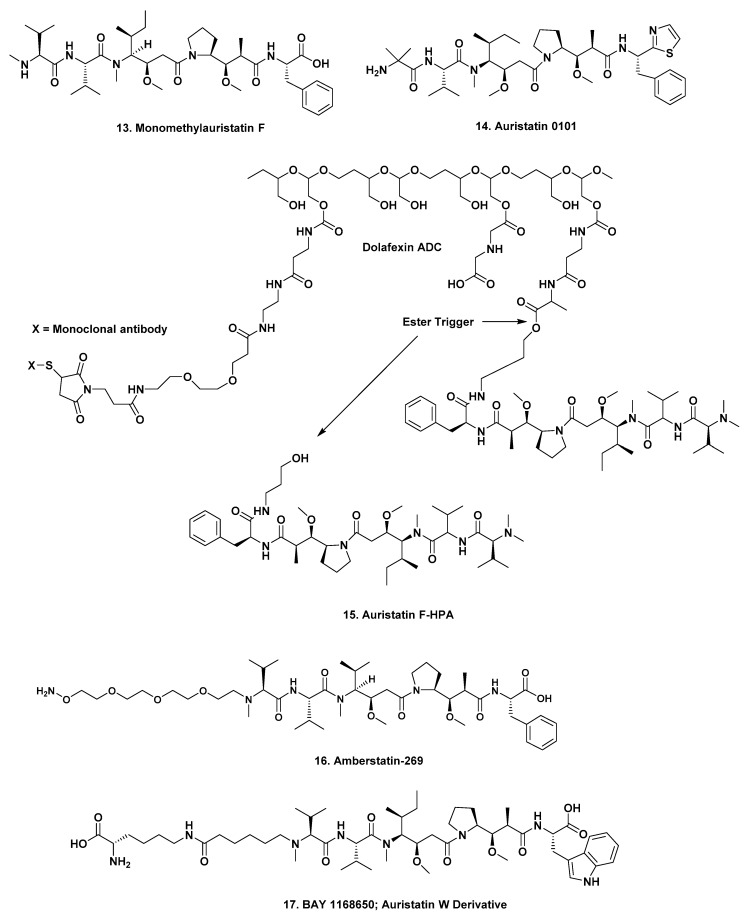
Structures **13** to **17**.

Almost all of the ADCs discussed in this part of the review, use a warhead that is based upon a derivative of the marine-sourced antitubulin agent dolastatin 10 (Figure 1; **1**). The dolastatins were originally isolated from the “Sea Hare” *Dolabella auricularia* and first reported in 1987 by the Pettit group [2]. There was also a concerted effort on the part of Genzyme scientists in the early 2000s to develop warheads around the linear dolastatin 15 molecule (Figure 1; **2**). This work led to an interesting report in 2012 by Gianolio et al. [3] where they used the basic structure of dolastatin 15 (Figure 1; **2**) as a warhead by synthesizing four variations that were designed to give different linkage sites at the N and C termini. Removal of the last two residues on the C-terminal end gave structure (Figure 1; **3**) which was designed with knowledge of the report by Pettit et al. in 1998 [4], demonstrating that replacement of the C-terminal (*S*)-dolapyrrolidinone unit by diverse amides maintained the anti-tubulin activities of these derivatives. 

**Figure 4:**
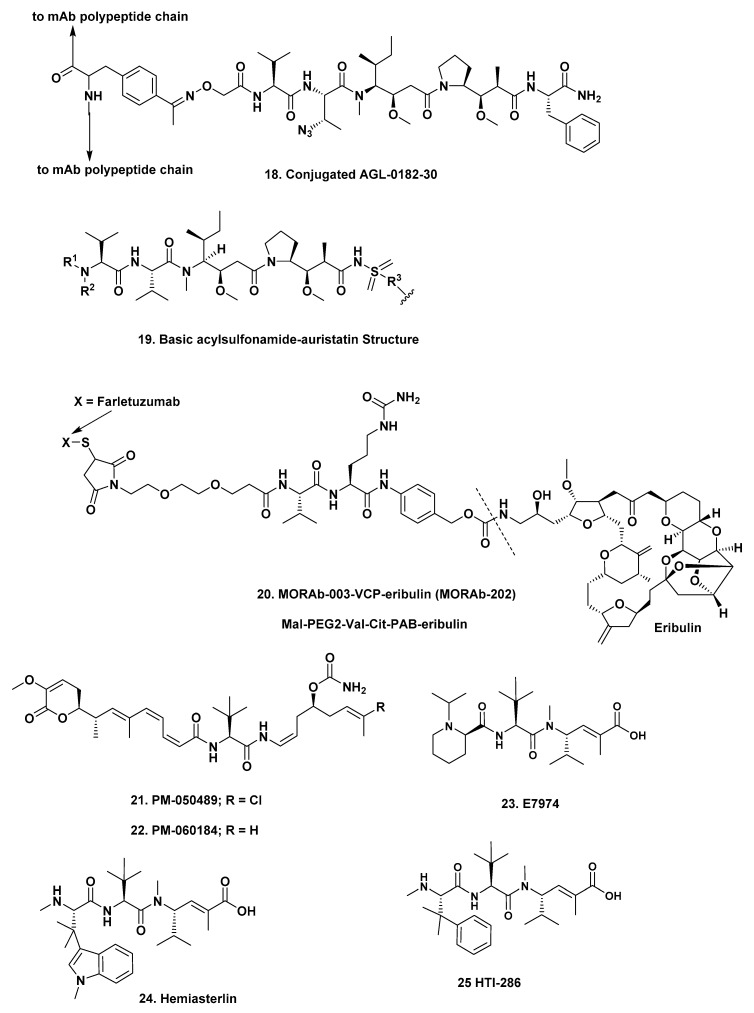
Structures **18** to **25**.

**Figure 5:**
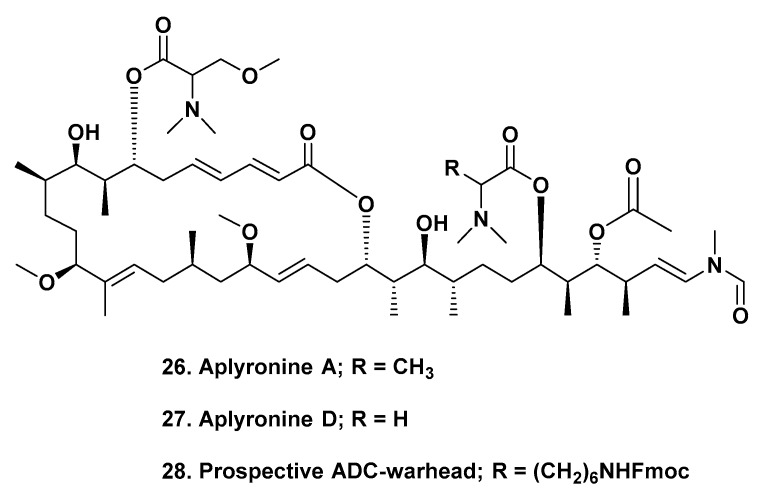
Structures **26** to **28**.

The Genzyme scientists then linked the N termini of such compounds to the mAb traztuzumab via a maleimidocaproyl (MC; Figure 1; 4) or a maleimido-caproyl-valine-citrulline-*p*-aminobenzyloxy-carbonyl linker (MC-VC-PABC; Figure 1; **5**) linking to partially reduced interchain disulfides on the traztuzumab mAb. When utilizing the C-termini, conjugates were formed either by esterification using structure (Figure 1; **6**), or by using a maleimide-based link (Figure 1; **7**). When C-terminus-linked, activity was demonstrated against *her2neu* expressing cells, but the corresponding N-terminal-linked ADC was inactive. Although the ester-linked variant (Figure 1; **6**) exhibited excellent in vivo activity when tested in SCID mice, no further work has been reported with these agents. The most significant finding however, was that dolastatin structures could be truncated and that such molecules had potential [3].

Rather than listing the significant number of papers that covered the early details of dolastatin 10 and its “natural relatives”, interested readers should consult both the original report by Pettit in 1987 [2] and his excellent review published in 1997 [5]. These covered the status at those times of this agent and “its many close natural relatives”. These articles covered isolation, purification and methods of synthesis, as the requirements for further testing made total synthesis the only feasible route. The story was continued up through 2011 in a later review by Flahive and Srirangam [6]. In the early 2000s, the true producer was shown to be a *Symploca* sp., a cyanophyte that the nudibranch grazed upon. Thus, the original marine-sourced compounds known collectively as the dolastatins, are all from prokaryotic sources [7].

In 1995, Pettit reported the syntheses of a variety of peptides related to dolastatin 10 [8,9]; then in 1998, following up on those earlier reports, he published a paper describing the results of further SAR studies on the basic dolastatin 10 structure [4] and further explained by Flahive and Srinangam [10]. Later, in 2011, Pettit’s group reported further modifications of the dolastatin 10 structure that possessed increased water solubility and maintained significant potency against tumor lines. Their activities were in the 10–100 pM range (IC_50_ values) depending upon the particular cell lines used [11]. These structures and syntheses of other modifications are given at length in the papers referred to above and include auristatin E (Figure 2; **8**), auristatin PHE (Figure 2; **9**), and auristatin PYE (Figure 2; **10**). 

Even though these materials were first reported in 1987, investigators are still interested in the basic skeleton, with more recent potential auristatins published in 2018 by Yokosaka et al. [12]—though nowadays, as mentioned in the title of that paper, the thrust is towards suitable agents for conjugation with mAbs.

It should be pointed out, that a number of compounds, including dolastatin 10, and some of the auristatins referred to above, did enter clinical trials as single agents against various cancers, but none proceeded beyond early Phase II trials. There were two trials at Phase II, and one at Phase I that was terminated before starting auristatin PE (as soblidotin). Tasidotin as ILX651, had three trials, all in the early 2000s with no reports at the Phase II level. Some of those early trials were cited by the author in papers published between 2004 and 2006, which gave further information as to their fates [13,14,15]. For Dolastatin 10 there was a Phase II trial in prostate cancer that demonstrated no efficacy [16], and a Phase II trial against advanced breast cancer in which, quoting from the authors, “it had minimal activity in this advanced breast cancer study. We are not pursuing further clinical trials of this agent in the setting of advanced breast cancer” [17]. That report was the last published data from a clinical trial of dolastatin 10 as a single agent.

## 3. Work on Dolastatin Derivatives as Warheads Leading to FDA Approval of Adcetris^®^

In 2003, Seattle Genetics scientists published an excellent paper on the early development of mAbs linked to both auristatin E (Figure 2; **8**) or a new analogue, monomethyl auristatin E (vedotin or MMAE) (Figure 2; **11**) with the base patent awarded in 2002 [18]. Seattle Genetics initially used a variety of linkers between the warhead and the mAb. Initially, an acid labile hydrazine linker was investigated and then an intermediate dipeptide (phenyl-lysine) or a valine-citrulline moiety linked to MMAE. Their experiments demonstrated that the latter two conjugation systems were more stable than the hydrazine-based linker. These later linkers used the amino terminus of the warhead and were compared with a C-terminal linker, the hydrazone of 5-benzoylvaleric acid-auristatin E ester (AEVB, structure not shown) [19,20]. 

These experiments over many years led to the identification, clinical trials and FDA approval of the first mAb-linked auristatin derivative, brentuximab vedotin (Adcetris^®^ (Figure 2; **12**)) in 2011, with an excellent report on its developmental history being published by Senter and Sievers of Seattle Genetics in 2012 [20]. 

In a paper published in 2015, scientists from Seattle Genetics discussed the site dependency of non-cleavable auristatin derivatives, which is relevant to earlier monomethyl auristatin F (MMAF (Figure 3; **13**)) conjugates. This article should also be read, as it covered the underlying rationales for choosing specific methodologies [21]. Further information that same year from the same group, discussed choices of cleavable sites [22]. Both of these 2015 papers followed on from an earlier paper published in 2013 by Seattle Genetics scientists with a different lead author, but with significant overlap of authors in all three papers [23]. Reading all three gives excellent insight into the problems involved and how they were overcome.

### 3.1. ADCs with Dolastatin-Derived Warheads in Clinical Trials as of Mid-April 2019

As of the time of writing there were 31 ADCs, not including brentuximab vedotin, in clinical trials at various stages ranging from an Investigational New Drug Application (IND) filed, to submission of New Drug Application (NDA) or equivalent Biologics License Application (BLA) to the relevant regulatory authorities (FDA or MEA). Of these, 19 are monomethyauristatin-E -linked (MMAE; (Figure 2; **11**)) in stages ranging from the INDA (which is requesting permission to begin clinical trials), to an NDA/BLA (submission of all clinical data to the FDA or equivalent for approval), with one at the IND stage, six at Phase I, four at Phase I/II, six at Phase II, one at Phase III and one (Polatuzumab vedotin) with an NDA/BLA applied for in the US (accelerated review as of 19 February 2019) and the EU. Three are monomethylauristatin F-linked, (MMAF (Figure 3; **13**)), with two at Phase II and one at Phase III. The remaining nine use modified auristatins, other than the basic MMAE or MMAF as the warheads, with one at Phase I/II, seven at Phase I and one preclinical. These are discussed separately. The number of agents at each phase is given as a number after the Phase heading.

### 3.2. Preclinical Auristatin-Based ADCs

Currently there are five ADCs that are in advanced preclinical status, of which three use MMAE, one uses a pegylated version of MMAE and one uses a fleximer^®^ linked Auristatin F derivative. These are listed under the respective major headings.

### 3.3. Discussion of Individual ADC Combinations from Approved to IND Status 

In each case, a relatively standard format is used, where the ADCs are subdivided by “warhead”, with a note given of the epitope(s), where known and/or reported, that the mAb is directed against. The number of clinical trials is, “as listed in the NIH clinical trials website as of the 16th of April 2019” that fall under the following categories: ”Not yet recruiting; Active but not recruiting; Recruiting by invitation; Recruiting”, are given, with their clinical trial phases; though if a trial has finished in the recent past (usually less than six months) it was noted as, “recruitment terminated and/or completed”. The latest trial is generally noted using the NCT number and title. This is different from the 2017 review where all were listed.

The identification of cancers that are being tested against will be normally be noted in trials at Phase I or above. It should be emphasized that Phase I cancer trials are conducted in patients with cancer, with the aim of generating information as to toxicity, dose tolerance, side effects, etc. In a regular Phase I trial (not using an ADC) sometimes there may be a positive response, but that is rare in such trials. With ADC’s, due to the specificity of the mAb used, patient cohorts will usually be selected at the Phase I level, with the cancers in the patient expressing the required epitope; examples would be in cancers expressing *Her2*, or a specific leukemia cell’s epitope.

It should be noted that a number is given after the phase heading, which notes the number of ADCs at that phase that will be discussed, with ADCs listed in alphabetical order under each phase listing, with the pharmaceutical company sponsoring the trial listed after the ADC’s identification code/name.

### 3.4. MMAE-Linked ADCs

#### 3.4.1. Approved: One

**Brentuximab vedotin** (Figure 2; **12**), *Seattle Genetics*, approved in the US in 2011. The mAb is a chimeric anti-CD30 antibody cAC10 (SGN-30) and is linked through a Val-Cit linker to four molecules of MMAE (Figure 2; **11**). Currently there are 72 studies at phases II to IV (61 at II, 8 at III, and 3 at IV). These include the Phase III trial NCT03907488, which includes immunotherapy by using nivolumab plus brentuximab vedotin, and combination chemotherapy as the standard of choice against newly diagnosed Stage II to IV classic Hodgkin’s Lymphoma in patients. Another of note, that is actively recruiting is NCT03138499, which is similar to the study above but compares nivolumab plus brentuxumab vedotin against just the ADC. The formal title is, “*A Study of Nivolumab Plus Brentuximab Vedotin Versus Brentuximab Vedotin Alone in Patients with Advanced Stage Classical Hodgkin Lymphoma, Who Are Relapsed/Refractory or Who Are Not Eligible for Autologous Stem Cell Transplant*”, (the CheckMate 812 Trial). To show the breadth of such a trial, even with a fully approved agent, there are 73 locations, including countries in the EU and Japan. 

Such trials are customary for approved antitumor drugs and to put it into perspective, the corresponding number for 5-Fluorouracil (5-FU), which was approved in the late 1950s, just at the Phase III level and only “recruiting or active but not recruiting”, is 150 studies with the largest proportion being in China (80 trials) followed by the EU at 72. As mentioned earlier, all of this information is freely available in the Clinicaltrials.gov website, and there are corresponding sites for the EU and other countries, particularly in the Far East.

#### 3.4.2. Pre-Registered: One

**Polatuzumab vedotin**, *Genentech*, is pre-registered with FDA accelerated review commencing in February 2019, with a decision possibly due by August 2019. The mAb is an anti-CD79 humanized monoclonal antibody conjugated to MMAE, with three to four molecules linked to cysteines via the cleavable maleimidecaproyl-valyl-citrullinyl-p-aminobenzyl -carbamate linker (mc-val-cit-PABC). There are currently eight ongoing trials; three at Phase I; four at Phase II and one at Phase III, NCT03274492—“*A Study Comparing the Efficacy and Safety of Polatuzumab Vedotin With Rituximab-Cyclophosphamide, Doxorubicin, and Prednisone (R-CHP) Versus Rituximab-Cyclophosphamide, Doxorubicin, Vincristine, and Prednisone (R-CHOP) in Participants with Diffuse Large B-Cell Lymphoma*”.

#### 3.4.3. Phase III: One

**Enfortumab vedotin**, *Seattle Genetics/Astellas Pharma*. The mAb is a fully human IgG1k monoclonal antibody that is specific for Nectin-4 and conjugated to MMAE via the valine-citrulline cleavable linker devised by Seattle Genetics. There are five current trials; two at Phase I, one at Phase I/II, one at Phase II and one at Phase III under NCT03474107—“*A Study to Evaluate Enfortumab Vedotin Versus Chemotherapy in Subjects with Previously Treated Locally Advanced or Metastatic Urothelial Cancer (EV-301)*”.

#### 3.4.4. Phase II: Six

**Ladiratuzumab vedotin**, *Seattle Genetics*. The mAb is a humanized hLIV22 antibody that targets human LIV-1, covalently linked to MMAE. LIV-1 is a member of the solute carrier family 39; a multi-span transmembrane protein with putative zinc transporter and metalloproteinase activity. LIV-1 expression has been linked to epidermal-to-mesenchymal transition (EMT) in both normal vertebrate embryo development and preclinical models, leading to malignant progression and metastasis [24]. This is currently in the Phase I/II trial NCT03310957—“*Safety and Efficacy of SGN-LIV1A Plus Pembrolizumab for Patients with Locally-Advanced or Metastatic Triple-Negative Breast Cancer*”. Since there are no effective treatments specifically for triple negative breast cancer, this will be an interesting ADC to follow.

**Lifastuzumab vedotin**, *Genentech*. The mAb is a humanized IgG1 monoclonal antibody targeting NaPi2b fused to MMAE. The “target” of the mAb is the anti-sodium-dependent phosphate transport protein 2B (also known as NaPi-2b or SLC34A2). When searching the NCT database, the original code number DNIB0600A has to be used. There is a published report of the results of the one (terminated) Phase II trial, NCT01991210—“*A Study of DNIB0600A in Comparison With Pegylated Liposomal Doxorubicin (PLD) in Participants With Platinum-Resistant Ovarian Cancer (PROC)*”—that was published by Banerjee et al. in 2018 [25]. Evidently this was the first trial of an ADC using a comparison against the standard of care in ovarian cancer patients. The trial was terminated due to adverse effects, though these were comparable to the PLD cohort, with promising responses to short-term use of the ADC. The authors commented that evaluation of both response rates and duration of response are necessary when testing ADCs in ovarian cancer.

**PSMA-ADC**, *Progenics*. The mAb is a fully human dimeric-specific PSMA monoclonal antibody conjugated to MMAE. Currently there are only “completed” trials shown in the clinical trials database, with two at Phase I and three at Phase II. The latest is NCT02020135 at Phase II, an extension trial with a small number of patients, but the earlier Phase II trial under NCT01695044 was directed against metastatic castration-resistant prostate cancer patients (mCRPC), with an abstract presented in 2015 at the Genitourinary Cancers Symposium [26]. The current status of this agent is uncertain as the Integrity^®^ database lists it as under development, but the Progenics web site has no mention of the agent as of mid-April 2019, and further searching showed a link to the ADIS Insight web site showing that the ADC was discontinued in 2018.

**RC-48**, *RemGen*. The mAb is a recombinant humanized anti-*Her2* monoclonal antibody-MMAE conjugate for the treatment of *Her2*-positive advanced breast cancer, urothelial cancer that is inoperable, locally advanced or metastatic, and gastric cancer that is locally advanced or metastatic in nature. Currently there are seven clinical trials using the criteria mentioned above, with two at Phase I, one at Phase I/II and four at Phase II. In Beijing, trial NCT03556345—“*A Study of RC48-ADC in Local Advanced or Metastatic Gastric Cancer Subjects with the Overexpression of* Her2”—is currently underway.

**Telisotuzumab vedotin**, *AbbVie*. The mAb is a humanized monoclonal IgG1 kappa antibody that targets hepatocyte growth factor receptor (HGFR) and is conjugated to approximately three molecules of MMAE. There are two Phase II trials underway: NCT03539536, “*Study of Telisotuzumab Vedotin (ABBV-399) in Subjects with Previously Treated c-Met+ Non-Small Cell Lung Cancer*” and NCT03574753, “*Lung-MAP S1400K; c-MET Positive*”.

**Tisotumab vedotin**, *GenMab/Seattle Genetics*. The mAb is a tissue factor-specific human IgG1k antibody conjugated to MMAE. Tissue factor is expressed in a variety of solid tumors including cervical cancer, epithelial ovarian cancer, colorectal cancer, squamous non-small cell lung cancer (NSCLC), pancreatic cancer, and squamous cell carcinoma of the head and neck (SCCHN). Currently there are seven clinical trials, with three at Phase I/II and four at Phase II, with the latest being NCT03485209 (I/II)—“*A Trial of Tisotumab Vedotin in Japanese Subjects with Advanced Solid Malignancies*”.

#### 3.4.5. Phase I/II: Four

**BA-3021** (also known as ROR2-CAB-ADC), *BioAlta*. The mAb is a humanized IgG1 antibody that is targeted against the tyrosine- protein kinase transmembrane receptor (ROR2), conjugated to MMAE. A Phase I/II trial is underway under NCT03504488, entitled, “*CAB-ROR2-ADC Safety and Efficacy Study in Patients with Solid Tumors*”.

**CX-2029**, *AbbVie*. Rather than a conventional immunoglobulin, this construct is a single polypeptide chain that contains the masking peptide, a protease cleavable linker and the antibody light chain, with the whole activatable system known as the Probody system^(TM)^ [27]. This system utilizes a CD71-targeting Probody^(TM)^ drug conjugated to MMAE and is being tested under NCT03543813—“*PROCLAIM-CX-2029: A Trial to Find Safe and Active Doses of an Investigational Drug CX-2029 for Patients with Solid Tumors or DLBCL*”.

**HuMax-AXL-107**, (also known as Enapotamab vedotin), *GenMab*. The mAb is a human monoclonal IgG1 antibody directed against Axl and conjugated to MMAE. Axl is a receptor tyrosine kinase (RTK) that belongs to the TAM (Tyro3, Axl and Mer) family [28]. There is one Phase I/II clinical trial underway, NCT02988817—“*HuMax-AXL-ADC Safety Study in Patients with Solid Tumors*”.

**Pinatuzumab vedotin**, *Genentech*. The mAb is a humanized immunoglobulin G1-Κ-anti-CD22 monoclonal antibody that is conjugated to three to four molecules of MMAE on average. There is one Phase I/II trial continuing under NCT01691898; a three-component study of, “*Pinatuzumab Vedotin (DCDT2980S) combined with Rituximab, or Polatuzumab Vedotin (DCDS4501A) Combined with Rituximab, or Obinutuzumab in Participants with Relapsed or Refractory B-Cell Non-Hodgkin’s Lymphoma*”. The ADC is listed as discontinued in the Genentech website, but the NCT still has it listed as “active but not recruiting”, and further searching in the NCT database implies that it is still an active trial, as the last update was posted in early January 2019. This is the type of discrepancy that can and does occur when data information is not compared in real time across databases. Pharmaceutical companies are supposed to update information in real time in the NCT database, but it does not always occur.

#### 3.4.6. Phase I: Six

**ABBV-085**, *AbbVie*. The mAb is directed against LRRC15, which is a novel mesenchymal protein and stromal target. LRRC 15 is membrane protein leucine-rich repeat that is expressed in multiple solid tumors and has low expression in normal tissue [29]. Currently one Phase I trial under NCT026565758 has been completed with the title, “*ABBV-085, an Antibody Drug Conjugate, in Subjects with Advanced Solid Tumors*”. The ultimate aims are to establish safety, pharmacodynamics and to derive a Phase II dose.

**AGS67E**, *Astellas/Seattle Genetics*. The mAb is a fully human anti-CD37 monoclonal IgG2 antibody conjugated to MMAE. Currently there is one Phase I trial NCT02175433—“*A Study to Evaluate Safety, Tolerability, and Pharmacokinetics of Escalating Doses of AGS67E Given as Monotherapy in Subjects with Refractory or Relapsed Lymphoid Malignancies*”. This is active but not recruiting. An earlier Phase I trial was terminated. There is a dichotomy in the literature as this ADC is not listed on the Astellas list of active trials (current as of January 2019) but is still listed in the NCT database as ongoing. Astellas has effectively wound down Agensys;it is therefore probable that this ADC will go no further.

**ALT-P7**, *3SBio/Alteogen*. The mAb is a trastuzumab variant (containing a metal-binding motif), that targets *Her2*, conjugated to MMAE. Currently there is a Phase I trial that is enrolling by invitation only, NCT03281824—“*Clinical Study of ALT-P7 to Determine Safety, Tolerability and Pharmacokinetics in Breast Cancer Patients*”.

**CDX-014**, *Celldex/Amgen*. The mAb is fully human anti- hepatitis A virus cellular receptor 1 (HAVCR-1/TIM1) monoclonal antibody coupled to MMAE. This construct targets the transmembrane protein (surface receptor) T-cell immunoglobulin mucin domain-1 (TIM-1). It is in early clinical trials for the treatment of renal cell and ovarian clear cell carcinomas, NCT02837991—“*A Dose Escalation, Safety and Activity Study of CDX-014 in Patients with Renal Cell Carcinoma and Ovarian Clear Cell Carcinoma*”. According to the NCT database, the trial was terminated in late November 2018, with a comment in the NCT database that “Development of CDX-014” was discontinued. To find this information it was necessary to search further in the NCT database, so it too, may have a similar status as Pinatuzumab vedotin above.

**Losatuxizumab vedotin**, (also known as ABBV-221) *AbbVie*. The mAb is a humanized and chimeric monoclonal IgG1 kappa antibody directed against the human epidermal growth factor receptor delta 2-7 isoform (delta2-7EGFR) and is conjugated to MMAE with a loading of approximately three molecules of MMAE on the mAb. The Phase I clinical trial NCT02365662—“*A Study Evaluating Safety and Pharmacokinetics of ABBV-221 in Subjects With Advanced Solid Tumor Types Likely to Exhibit Elevated Levels of Epidermal Growth Factor Receptor*”—is now listed as terminated, and the compound is no longer listed in the AbbVie web site under either name. A recent publication does provide data on the characterization of this ADC [30]. The actual status of this particular ADC is debatable, as with the two referred to above and demonstrates that searchers have to use more than one source for accurate and up to date information.

**SGN-CD48A**. *Seattle Genetics*. The mAb is a humanized antibody targeted against the CD48 epitope conjugated to MMAE. Currently there is one Phase I trial under NCT03379584—“*A Safety Study of SGN-CD48A in Patients with Multiple Myeloma*”.

#### 3.4.7. IND Filed: One

**Rituximab-MC-vc-MMAE; TRS-005**, *Zhejiang Teruisi Pharmaceutical*. The mAb is the chimeric monoclonal antibody rituximab that targets CD- 20 conjugated to MMAE with a drug-antibody ratio of 4:2. Thus, it is directed towards non-Hodgkin’s lymphoma at this moment.

#### 3.4.8. Advanced Preclinical: Four

*Bioatla*. This has no code number or name at the moment, but the FDA gave an Orphan Drug Designation on 19th November 2018, for a humanized IgG1 antibody that is directed to the AXL tyrosine kinase conjugated to MMAE. The company intends to develop this agent against pancreatic cancer.

**GM-103**. *Gamma Mabs Pharma*. The mAb is a humanized IgG1 low fucose 2C23K antibody, that is designed to target the anti-Müllerian hormone type II receptor (AMHR-II) conjugated to MMAE.

**HT-1511**. *Halozyme*. The mAb is a proprietary HALO monoclonal antibody that targets EGFR and is conjugated to MMAE via a short branched-chain PEG spacer. The drug-antibody ratio is four, and significant in vivo activity was seen in murine models with significant over-expression of EGFR MDA-MB-231M (triple negative breast cancer) and HT-29 (colorectal cancer with BRAF-V600E) [31].

**OBI-999**. *OBI Pharma*. The mAb is designed to target globohexaosylceramide (globo-H) covalently linked to MMAE with a DAR ratio of four.

### 3.5. MMAF-Linked ADCs

#### 3.5.1. Phase III: One

**Depatuxizumab mafadotin**, *AbbVie*. The mAb is ABT-806, an anti-EGFR humanized IgG1 monoclonal antibody conjugated to MMAF (Figure 3; **13**) using a non-cleavable linker. Currently, there are four trials that fit the requirements listed earlier;ne at Phase I, two at Phase II/III and one at Phase III, under NCT03419403 UNITE Study—“*Understanding New Interventions with GBM ThErapy*”. A recent (2019) publication provided data on Phase I studies against glioblastoma multiforme with this agent, and the Phase III trial is directed against this cancer, for which there are very limited treatments [32].

#### 3.5.2. Phase II: Two

**AGS-16C3F**, *Astellas Pharma*. The mAb is a fully human IgG2k monoclonal antibody that targets the AGS-16 antigen and is conjugated to MMAF using a non-cleavable linker. Currently there is one Phase II trial underway under NCT02639182—*A Study of AGS-16C3F vs. Axitinib in Metastatic Renal Cell Carcinoma*”. Interestingly, two sources were initially used to develop the mAb, but the Chinese hamster ovary-based mAb became the one chosen [33].

**GSK-2857916, Belantamab mafodotin**, *GSK*. The mAb is an anti-BCMA afucosylated humanized monoclonal antibody (code number J6M0) conjugated to MMAF. It is directed against patients with multiple myeloma who failed three or more prior treatments, including any related to a CD33-antibody. In 2017, the EU granted PRIME designation, and that year the FDA gave it a breakthrough designation. Currently there are six trials underway, two at Phase I, one at Phase I/II and three at Phase II, with the latest being NCT03848845—“*Study Evaluating Safety, Tolerability and Clinical Activity of GSK2857916 in Combination with Pembrolizumab in Subjects with Relapsed/Refractory Multiple Myeloma (RRMM)*”. Recently a report on one of the earlier Phase I trials was published, demonstrating significant efficacy and safety in an expanded Phase I study [34]. 

### 3.6. Modified Auristatins as Warheads

Over the years since Seattle Genetics revealed the utility of structures of MMAE and MMAF, chemists have been modifying not only those molecules, but also working with the earlier variations on dolastatin 10 as potential warheads for a variety of ADCs that are now in clinical trials. In this section, each variation currently in clinical trials is discussed with the structures shown as far as can be determined from a variety of sources, with references if available. 

#### 3.6.1. Auristatin 0101

##### Phase I/II: One

**W-0101**, *Pierre Fabre*. The mAb in this construct is designed to bind to cell types that overexpress the insulin-like growth factor type 1 receptor (IGF-1R) and is coupled to auristatin-0101 (Figure 3; **14**). Preliminary data in mice were presented at the 2018 AACR Meeting in the form of an abstract [35], and currently the construct is in a Phase I/II trial under NCT03316638—“*A Research Study of a New Investigational Medicinal Product for the Treatment of Patients With Advanced or Metastatic Solid Tumors*—that is actively recruiting. It should be pointed out, that Pierre Fabre have not formally shown the structure of A-0101 but other ADCs have used the same code number with the same structure that is shown in Figure 3.

##### Phase I: Two

**Cofetuzumab pelidotin**. *Pfizer/AbbVie*. This is also known as PF-06647020. The mAb is a humanized monoclonal antibody, initially from the Jackson laboratories, and coded as h6M24, directed against anti-tyrosine-protein kinase 7 (PTK7), and is conjugated using a cleavable linker to auristatin-0101 (Figure 3; **14**). There are two Phase I trials currently shown in the NCT databases, with one (NCT02222922) that is still active but not recruiting and is due to finish within a month. The more recent one, that is in the recruiting stage, is NCT03243331 entitled, “*An Initial Safety Study of Gedatolisib Plus PTK7-ADC for Metastatic Triple-negative Breast Cancer*”. It should be noted that the trial listings are under the PF “number” not the generic name. 

**NG-HER2 ADC** (also known as PF-06804103), *Pfizer*. The mAb is directed against the human epidermal growth factor receptor (*Her2*) and is linked to auristatin-0101 using a protease cleavable linker and is almost certainly a similar system to that used for Cofetuzumab pelidotin (PF-06647020). The current trial under NCT03284723 is entitled, “*PF-06804103 Dose Escalation in HER2 Positive Solid Tumors*” and is actively recruiting. Pfizer scientists reported a mouse study comparing the approved ADC, trastuzumab emtansine, against this construct in an abstract presented at the 2018 AACR Meeting in Chicago [36].

#### 3.6.2. Auristatin F-HPA

##### Phase I: One

**XMT-1536**. *Mersana*. The mAb targets the sodium-dependent phosphate transport protein 2B (NaPi2b) and is conjugated to auristatin F-HPA (Figure 3; **15**) through a flexible polymer (Dolaflexin^TM^) and has a drug to antibody ratio of 15:1. There is an excellent description of the process involved with this agent given in a published AACR Poster by Clardy et al. in 2018 [37]. The abstract contains the following description of the process:

“The controlled bystander effect, termed “DolaLock”, was achieved through design of a payload, auristatin F-hydroxypropylamide (AF-HPA) {**15**}, that is membrane-permeable and capable of bystander killing but is further catabolized to membrane-impermeable auristatin F (AF). This catabolism of the payload “locks” the highly potent AF in the cell. Using Dolaflexin-based ADCs, we investigated the extent of intracellular AF-HPA and AF release, tumor cell retention and bystander activities in vitro and in vivo. We observed both auristatin species within cells. Co-culture assays with HER2-positive and HER2-negative cells confirmed the cell permeability and bystander killing capabilities of AF-HPA released from a Dolaflexin-based ADC. Biodistribution studies of Dolaflexin-based ADCs revealed time-dependent concentrations of AF-HPA and AF as well as significant accumulation of AF in xenografted tumor cells, consistent with the Dolalock mechanism. An additional benefit of AF formation was seen in multidrug resistant transporter studies which demonstrate that AF, in contrast to AF-HPA, is not a P-glycoprotein 1 (Pgp) substrate. This property may offer additional benefit in Pgp-expressing tumors.”

Currently, there is one Phase I trial recruiting under NCT03319628, entitled, “*First-in-Human Study of XMT-1536 in Cancers Likely to Express NaPi2b*”, directed against platinum-resistant ovarian cancer, non-small cell metastatic lung cancer and papillary renal cell carcinoma. The points of scission are shown in Figure 3 below.

##### Preclinical: One

**ASN-004,***Mersana*. The mAb a humanized scFvFc antibody that targets the trophoblast glycoprotein (5T4) and is conjugated via the “Dolaflexin system” to auristatin F-HPA (Figure 3; **15**) with a drug to antibody ratio of 15. Also known as the Fleximer system. The same description was given for XMT-1536 above, so it is highly probable that this is the same warhead [38].

#### 3.6.3. Amberstatin 269 

##### Phase I: One

**ARX-788**. *Zhejiang Medicine/Ambrx*. The antibody is *Her2*-specific conjugated to amberstatin 269 (Figure 3; **16**), that is in two Phase I trials against adult *Her2* positive metastatic breast cancer. Both trials are actively recruiting patients, with the latest one being NCT03255070—“*A Dose-escalation Study of ARX788, IV Administered in Subjects With Advanced Cancers With HER2 Expression*”. Amberstatin 269 (Figure 3; **16**) is a derivative of MMAF with a polyethylene glycol chain at the N-terminus, that is then linked to the mAb.

#### 3.6.4. Auristatin W Derivative 

##### Phase I: One

**Lupartumab amadotin** (also known as Bay1129980), *Bayer*. The mAb is a human–human monoclonal immunoglobulin IgG1 lambda1 anti-human LYPD3. This is a protein coding gene that is a GPI-anchored cell surface protein C4.4a, C4.4A linked to metastasis and conjugated to an auristatin W derivative (Figure 3; **17**). According to both the Seattle Genetics and the Bayer websites, this construct is still in Phase I, but the relevant NCT trial listing (NCT02134197—“*Dose-escalation Study of Lupartumab Amadotin (BAY1129980)*”—is currently, as of mid-April 2019, listed as “recruitment terminated”. Since this trial was scheduled to finish on 30th August 2018 further trials may well be forthcoming.

#### 3.6.5. AGD-0182

##### Phase I: One

**AGS-62P1**. *Astellas*. The mAb is a human monoclonal IgG1 antibody that is targeted against FLT3. This mAb is conjugated to an azide-containing cytotoxic drug through an oxime bond at a *p*-acetyl phenylalanine site, with a drug-antibody ratio of close to two. The warhead uses the code name AGL-0182-30 and contains a variation on MMAF (AGL-0182-30; (Figure 4; **18**)) that has a non-cleavable linker. Details of the ADC, including its novel oxime linker, and its formal degradation in vivo were recently published by Snyder et al. [39]. Currently this ADC is in one Phase I study against acute myeloid leukemia, under NCT02864290—“*A Study to Evaluate Escalating Doses of ASP1235 (AGS62P1) Given as Monotherapy in Subjects with Acute Myeloid Leukemia (AML)*”.

#### 3.6.6. *N*-acylsulfonamide-Auristatin

##### Phase I: One

**ZW-49**. *Zymeworks*. The mAb in this construct is an antibody that targets *Her2* and is linked via a proprietary N-acyl-sulfonamide-auristatin molecule. It is currently in a Phase I study under NCT03821233—“*A Dose Finding Study of ZW49 in Patients with HER2-Positive Cancers*”—with patients being actively recruited. The actual structure of the “warhead” has not been formally divulged, but a search of the literature yielded two abstracts presented in 2018 [40,41], which when coupled to information contained in a patent application (WO 2015/095953A1) yielded the basic structure shown (Figure 4; **19**), a variation on MMAE. The website of Zymeworks implies that there are other potential warheads also under development.

### 3.7. Non-Auristatin-Based

#### 3.7.1. Eribulin

##### Phase I: One

**MORAb-003-VCP-eribulin (MORAb-202)**, *Eisai*. In an excellent recent paper, Eisai scientists reported on their work that led to the synthesis and choice of one ADC that used the halichondrin B based molecule eribulin as its warhead (Figure 4; **20**) [42]. The mAb in this case is known as farletuzumab and is a humanized anti-human antibody directed against the FRα receptor with an antibody to warhead ratio of four. Currently this construct is in a Phase I trial, under the auspices of Eisai in Japan, against solid tumors. The relevant code number is not available at the time of writing.

#### 3.7.2. Preclinical: Two

**PM-050489**, *PharmaMar*. This ADC uses the PharmaMar compound PM-050489 (Figure 4; **21**), the naturally occurring chlorinated derivative of PM-060184 (Figure 4; **22**) which is in Phase II clinical trials as a single agent under the code number PM-184. The ADC consists of PM-050489 conjugated to the mAb trastuzumab via a maleimidocaproyl (mc) linker under the code MI-130004. This ADC is designed for treatment of *Her2*-expressing tumors, including breast, gastric and ovarian carcinomas.

**NC-6201**, *Eisai*. This is a somewhat unusual construct as it is composed of antibody-drug conjugate micelles. In this construct, the mAb NCAB001 that targets EGFR, is attached to nanoparticles composed of self-assembling maleimide-PEG-poly-(glutamic acid benzyl ester) micelles entrapping E-7974 (Figure 4; **23**), which is a derivative of the marine-derived tripeptide, hemiasterlin (Figure 4; **24**) with E-7974 being covalently linked to a PEG-poly(glutamic acid benzyl ester. The biological activity of E-7974 was initially described by Eisai scientists in 2009 [43]. Another derivative of hemiasterlin known as HTI-286 (Taltobulin) (Figure 4; **25**), synthesized by the Andersen group who first reported hemiasterlin, was a Phase II candidate as a single agent against cancer under Wyeth, and was cancelled for “business reasons” [44], in the mid-2000s.

## 4. Potential Warheads from Marine Sources, Aplyronines A and D

The very potent compound series from marine sources known as the aplyronines could well have significant potential as a source of warheads. These compounds are dual tubulin and actin inhibitors, thus complementing the dolastatin-derived tubulin inhibitors described above. A very considerable amount of work was performed in the 1990s by the Japanese group that discovered the series, showing that in in vivo tests against murine tumors in normal mice conducted in Japan, using materials isolated from *Aplysia kurodai*, significant activity was seen with the A congener. Later work demonstrated significant activity in the NCI’s 60 cell line panel. Even though the Japanese group had to synthesize the compounds in the series due to their very low natural abundance, similar to what Pettit had to do with the dolastatins, their syntheses were not significantly scalable. 

Recently, Paterson’s group at Cambridge University in the UK published a synthesis that has the potential to produce sufficient quantities of both aplyronines A (Figure 5; **26**) and D (Figure 5; **27**) to be useful as warhead candidates. They also suggested a simple modification (Figure 5; **28**) designed to link the base molecule(s), via a peptide bridge, to suitable intermediates such as side-chains on a mAb, or via or another linker that is attached to the mAb [45]. Since these agents are highly toxic with defined mechanisms of action, these suggestions are definite possibilities.

## 5. Conclusions

As can be seen from the details shown above, highly toxic molecules isolated from marine sources have significant possibilities as “warheads” for very specific monoclonal antibodies. Currently there are four approved ADCs in clinical use, though only one at this moment is from a marine-sourced molecule. However, what is of scientific interest is that all current approved ADCs utilize microbial products as their warheads. 

Though over 35 ADCs have been considered in this review, there are currently more than 100 in some form of clinical trial. Some of the other 60 plus ADCs may well use a variation on a marine natural product but due to the pharmaceutical houses not releasing their “proprietary warhead structures” even when compounds are in Phase I/II trials, the actual numbers cannot be determined. Due to some inconsistences among databases and company websites, there are at least three of the ADCs covered that may no longer be in development. Unfortunately, there is no absolute measure of compounds in a company’s developmental pipeline that is externally accessible, therefore, where there was a dichotomy in results, it was mentioned. However, in spite of such problems, the utility of highly toxic marine-sourced molecules is still apparent, though not in the manner that the original discoverers would have envisaged.
